# Pharmacological Features of 18β-Glycyrrhetinic Acid: A Pentacyclic Triterpenoid of Therapeutic Potential

**DOI:** 10.3390/plants12051086

**Published:** 2023-03-01

**Authors:** Pottathil Shinu, Girdhari Lal Gupta, Manu Sharma, Shahzad Khan, Manoj Goyal, Anroop B. Nair, Manish Kumar, Wafaa E. Soliman, Aminur Rahman, Mahesh Attimarad, Katharigatta N. Venugopala, Abdullah Abdulhamid Ahmed Altaweel

**Affiliations:** 1 Department of Biomedical Sciences, College of Clinical Pharmacy, King Faisal University, Al-Ahsa 31982, Saudi Arabia; 2 Department of Pharmacology, School of Pharmacy and Technology Management, SVKM’s NMIMS University, Shirpur 425405, India; 3 Department of Chemistry, National Forensic Sciences University Delhi Campus, New Delhi 110085, India; 4 Department of Anesthesia Technology, College of Applied Medical Sciences in Jubail, Imam Abdul Rahman Bin Faisal University, Jubail 35816, Saudi Arabia; 5 Department of Pharmaceutical Sciences, College of Clinical Pharmacy, King Faisal University, Al-Ahsa 31982, Saudi Arabia; 6 Department of Pharmaceutics, M. M. College of Pharmacy, Maharishi Markandeshwar (Deemed to Be University), Ambala 133201, India; 7 Department of Microbiology and Immunology, Faculty of Pharmacy, Delta University for Science and Technology, Mansoura 11152, Egypt; 8 Department of Biotechnology and Food Science, Faculty of Applied Sciences, Durban University of Technology, Durban 4000, South Africa

**Keywords:** 18β-glycyrrhetinic acid, licorice, pharmacological action, triterpenoid

## Abstract

*Glycyrrhiza glabra* L. (belonging to the family Leguminosae), commonly known as Licorice, is a popular medicinal plant that has been used in traditional medicine worldwide for its ethnopharmacological efficacy in treating several ailments. Natural herbal substances with strong biological activity have recently received much attention. The main metabolite of glycyrrhizic acid is 18β-glycyrrhetinic acid (18βGA), a pentacyclic triterpene. A major active plant component derived from licorice root, 18βGA has sparked a lot of attention due to its pharmacological properties. The current review thoroughly examines the literature on 18βGA, a major active plant component obtained from *Glycyrrhiza glabra* L. The current work provides insight into the pharmacological activities of 18βGA and the potential mechanisms of action involved. The plant contains a variety of phytoconstituents such as 18βGA, which has a variety of biological effects including antiasthmatic, hepatoprotective, anticancer, nephroprotective, antidiabetic, antileishmanial, antiviral, antibacterial, antipsoriasis, antiosteoporosis, antiepileptic, antiarrhythmic, and anti-inflammatory, and is also useful in the management of pulmonary arterial hypertension, antipsychotic-induced hyperprolactinemia, and cerebral ischemia. This review examines research on the pharmacological characteristics of 18βGA throughout recent decades to demonstrate its therapeutic potential and any gaps that may exist, presenting possibilities for future drug research and development.

## 1. Introduction

Plants have long been employed as an essential source of medicine in all cultures. Various indigenous herbs are utilized to prevent and eradicate acute and chronic illnesses in the traditional system. Herbal medications, health products, and pharmaceuticals are in high demand throughout the globe, since they are safe, effective, and culturally acceptable and have fewer adverse effects [1]. Medicinal plants have become an important aspect of contemporary life as a source of therapeutic assistance for a variety of human ailments. Research on a global scale has been conducted to investigate how effective they are, and some of the findings have contributed to the creation of medicines derived from plants [2]. Many drugs from Glycyrrhiza species (Fabaceae) have been utilized in folk medicine worldwide for their ethnopharmacological usefulness in treating various illnesses. *Glycyrrhiza glabra* L., *Glycyrrhiza uralensis* Fisch., and *Glycyrrhiza inflata* Batalin plant names have been verified with http://www.theplantlist.org (accessed on 21 January 2022), and plant name is the recognized name of a species in the genus Glycyrrhiza [3]. The primary therapeutic parts of Glycyrrhiza are the roots and rhizomes [4]. Licorice, mulaithi, and yashtimadu are all names for *Glycyrrhiza glabra* L. *Glycyrrhiza glabra* L. gets its name from the Greek words *glykos* (sweet) and *rhiza* (root). Because of its sweetness, this herb’s roots are frequently used as a flavoring ingredient. *Glycyrrhiza glabra* L. roots are an essential ingredient in Indian traditional medicine systems and function as ulcer protectants, demulcents, expectorants, and antitussives [4,5]. The genus Glycyrrhiza includes approximately 30 species that are found all over the globe. Plants of the Glycyrrhiza genus are found throughout Europe, Asia, the UK, the USA, China, Asia, and the Mediterranean basin. Commercial growth on a large scale is reported in Spain, Sicily, and England [5,6]. 

**Figure 1:**
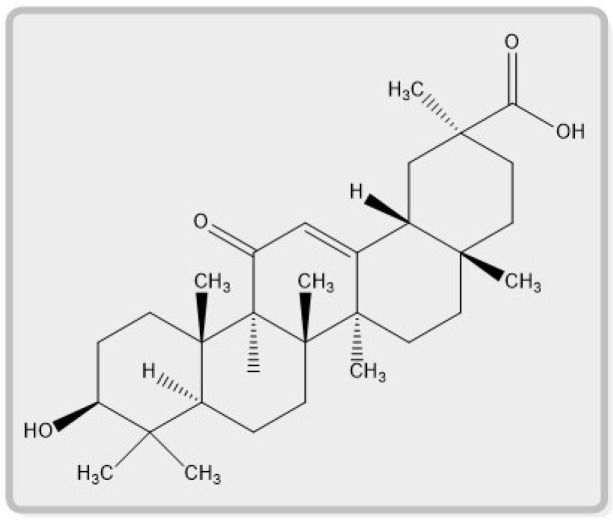
The chemical structure of 18β-glycyrrhetinic acid.

Furthermore, several published studies describe the various secondary metabolites in Glycyrrhiza species. *Glycyrrhiza glabra* L. contains more than 20 types of triterpenoids and over 300 flavonoids. The main sweet-tasting component of *Glycyrrhiza glabra* L. root is glycyrrhizin. It is a saponin employed as an emulsifying agent in foods and cosmetics [7]. When ingested, glycyrrhizin itself is not well absorbed. Bacteria in the digestive tract break it down to the aglycone, glycyrrhetic acid, which is rapidly absorbed and has pharmacological actions. Glycyrrhiza species comprise various chemical components, including coumarins, stilbenoids, saponins, and polyphenols. The major active constituents of *Glycyrrhiza glabra* L. root are triterpenoid saponins such as glycyrrhizin. These are the compounds that give licorice its distinctively sweet flavor. Liquiritic acid, glycyrretol, glabrolide, uralsaponin B, apioglycyrrhizin, araboglycyrrhizin, and licorice acid are examples of additional triterpenes. Glycyrrhizic acid has two aglycone forms, 18β-glycyrrhetinic acid and 18α-glycyrrhetinic acid. In flavonoids, the active components include liquirtin, rhamnoliquirilin, shinflavanone, liquiritigenin, apioside, and neoliquiritin. Glabridin, glabrone, glyzarin, and galbrene are present as isoflavonoids. Liqcoumarin, iqcoumarin, glabrocoumarone A and B, herniarin, umbelliferone, glycocoumarin, and umbelliferone are the active components in coumarins. Glycyrrhetinic acid is produced when a saponin glycoside is hydrolyzed. The most significant (10 to 25 percent) active component of *Glycyrrhiza glabra* L. root extract is glycyrrhizin, sometimes referred to as glycyrrhizic acid [8]. The chemical structure of 18β-glycyrrhetinic acid is described in Figure 1.

From licorice, two stereoisomers of glycyrrhetinic acid were isolated: 18βGA and 18α-glycyrrhetinic acid. Glycyrrhetinic acid is an oleanane-type triterpenoid with a carboxylic acid instead of a methyl group at C-30. Furthermore, the quantity of 18βGA in licorice root ranges from 0.1 to 1.6 percent, while 18α-glycyrrhetinic acid is usually less than 0.7 percent [9]. After metabolism in the plant and human intestine, 18β-glycyrrhizin, a key component of licorice, degrades into pentacyclic triterpenoid 18βGA [4]. Moreover, 18α-Glycyrrhizin is an epimer of 18β-glycyrrhizin, the minor ingredient of licorice [10]. The pentacyclic triterpenoids, which have a basic chemical structure with five rings, have been very interesting because of their pharmacological effects [11]. As a result, 18βGA and its derivatives have various pharmacological effects and, most important, naturally occurring compounds [12]. 

**Figure 2:**
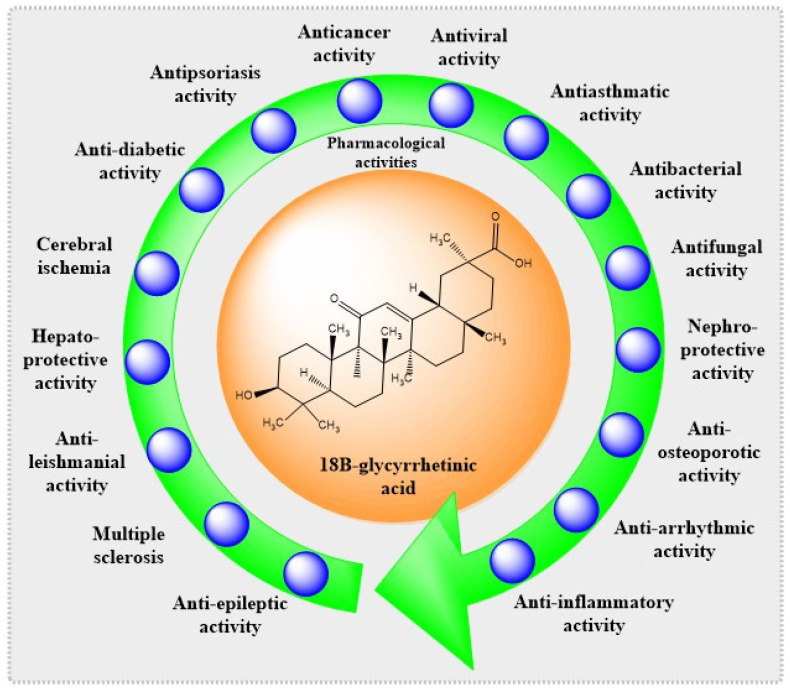
The pharmacological activities of 18β-glycyrrhetinic acid.

Enoxolone, glycyrrhetin, 3β-hydroxy-11oxo-18β, 20β-olean12-en-29oic acid, uralenic acid, and sub-glycyrrhetinic acid are all names for 18βGA. Based on preclinical findings, 18βGA is recognized as having antiasthmatic, hepatoprotective, anticancer, nephroprotective, antidiabetic, antileishmanial, antiviral, antibacterial, antifungal, antipsoriasis, antiosteoporotic, antiepileptic, antiarrhythmic, neuroprotective, and anti-inflammatory properties and is also beneficial in managing cerebral ischemia, pulmonary arterial hypertension, and antipsychotic-induced hyperprolactinemia, as described in Figure 2.

The glycyrrhetinic acid scaffold has undergone significant chemical alterations, as seen by the multiple patents submitted between 2010 and 2017. A substantial number of novel GA analogs are now available for biological testing. Amidation or esterification of the -COOH moiety at C-30 in ring-E, the addition of a cyano group at the C-2 position, and an additional hydroxyl group to GA are all important ways to enhance glycyrrhetinic acid’s pharmacological actions, especially cytotoxic effects [9]. The effects of anticancer, antimicrobial, anti-inflammatory, antioxidant, painkiller, and antiviral glycyrrhetinic acid analogs have been shown in a study of the patents issued concerning natural and synthetic analogs from January 2010 to December 2017 [9]. Moreover, our review examines recent research on the pharmacological characteristics of 18βGA throughout recent decades to demonstrate its therapeutic potential and any gaps that may exist, presenting possibilities for future drug research and development. Table 1, Table 2, Table 3 and Table 4 summarize the model, dose, pharmacological effects, and mechanism of action of 18βGA.

## 2. Selection of the Literature

The following resources were thoroughly examined for pertinent English-language articles between 2012 and 2022: PubMed, Scopus, Web of Science, Science Direct, Google Scholar, and Medline. However, few works conducted before 2012 were included in the introduction and explanation of context. For our literature search, we utilized the terms “18β-glycyrrhetinic acid” alone or in combination with “licorice” and “chemical aspects and biological activities.” The articles on biosynthesis methods, derivatives of 18β-glycyrrhetinic acid, and 18α-glycyrrhetinic acid were excluded from the study. After reading the titles, abstracts, and full reports, around 110 papers about phytochemical research and pharmacological effects of 18βGA were finally included.

### Bioactive Derivatives of 18β-Glycyrrhetinic Acid

Pentacyclic triterpenoids are physiologically beneficial natural compounds that have been extensively studied for their fascinating medicinal and pharmacological activities. Glycyrrhetinic acid is a remarkable example of skeletal chemical variation, since it has a C-3 hydroxyl group, a C-11 keto moiety, and a C-30 carboxylic acid. This makes it a good candidate for the study. It has been shown that the hydroxyl group at position C-3 is essential for preserving the cytotoxicity of glycyrrhetinic acid [13,14]. In addition, an extra amino group, amino acid, or nitrogen-comprising group was necessary for the cytotoxic effects to be enhanced. However, the activities were significantly reduced when the C-3 hydroxyl group was changed to the keto group. Several different substituents are essential for improving the cytotoxic effects of the compound, particularly in ring A. The cytotoxic effects of ring A were significantly amplified when either a cyano (CN) or trifluoromethyl (CF3) group was introduced at position C-2. The production of GA derivatives containing enones in ring A, notably cyanoenones, as well as the introduction of heterocyclic ring systems and amino-comprising alkyl groups at positions C-3 and C-30 should be the primary focus of research to be conducted in the future [14,15].

The cost-to-benefit ratio makes difficult the delivery of large quantities of GA to the chemical and pharmaceutical industries. Any chemical conversion or medicinal use of GA derivatives is known to rely on raw material availability. As a result, solutions for the extraction and purification of GA must be developed that are productive, economically feasible, efficient, and environmentally benign.

Furthermore, owing to its low bioavailability and hydrophobicity, GA’s general application in cancer therapy is severely limited. Various GA-synthesized compounds with exocyclic-unsaturated carbonyl groups in ring A and nitrogen-containing polar groups such as aniline and 4-aminopyridine have been produced. The polar groups placed into the GA skeleton or conjugated with GA efficiently increase the solubility and bioavailability of GA synthetic derivatives [16].

Glycyrrhetinic acid endoperoxide derivative shown to have strong cytotoxic effects on A2780, and MCF7 cells, with IC_50_ values ranging from 1.0 (A2780) to 2.9 (MCF7). Glycyrrhetinic acid analog with an extra hydroxyl group at C-3 also exhibited significant cytotoxic effects with an IC_50_ of 0.22 µM against the HepG-2 cancer cell line. Gao et al. synthesized glycyrrhetinic acid analogs that all had cytotoxic effects against leukemia cells (HL-60), with IC_50_ values ranging from 1.7 to 8.6 µM.

## 3. Pharmacological Properties of 18β-Glycyrrhetinic Acid

Natural products have characteristics that distinguish them from synthetic compounds, providing benefits and challenges throughout the drug development process.

### 3.1. Anticancer Activity

Despite significant advancements in research, cancer continues to be the main reason for mortality. Cancer is the second leading cause of mortality after heart disease. According to statistics, cancer is responsible for roughly 23% of fatalities in the United States. As a consequence, new ways of combating the disease must be devised. Saponins are a kind of natural chemical that has been shown to have strong anticancer properties. Several reports exist on the use of saponin glycoside against various cancer kinds and targets. Glycyrrhizae radix, the most often used element in Chinese medicine formulae, has been utilized to cure ailments (including cancer) in China for thousands of years. The influence of 18βGA on several cancer types has been documented here.

#### 3.1.1. Breast Cancer 

One of the most frequent causes of death among women globally is breast cancer, which is also the top killer of women. Further, the primary conventional surgical procedure is no longer the best option for all patients [17]. Proteolytic enzymes degrade the extracellular matrix in the first phases of cancer metastasis, followed by cell migration. The matrix metalloproteinase (MMP) family plays a key role in these processes. MMP-2 and MMP-9 expression/activities typically increase in metastatic cancer types such as breast, colon, and lung. The increased MMP-2/9 activity/expression in breast cancer cells needs constitutive p38 MAPK activation. These bioactive chemicals have been reported to unleash therapeutic properties by lowering antitumor medication adverse effects or directly killing cancer cells [18]. Interestingly, Wang et al. (2015) demonstrated that 18βGA inhibits the p38 mitogen-activated protein kinase (MAPK)-AP1 signaling axis, inhibiting breast cancer cell invasion and breast tumor development pulmonary metastasis [19]. Hence, 18βGA might be a promising candidate for evolving a therapeutic breast cancer drug (Table 1).

**Table 1:** Hepatoprotective, anticancer activity, role in arrhythmia, ischemic stroke, and mechanisms of 18β-glycyrrhetinic acid in both in vitro and in vivo.

Assay	Model	Dose/Concentration of 18β-Glycyrrhetinic Acid	Effect	Mechanisms	Reference
In vivo	Wistar rats	50 and 100 mg/kg, oral for 7 days	hepatoprotective activity	Downregulation of PPARγ and Nrf2	[20]
In vivo and in vitro	Male Sprague Dawley rats and HEK293T cells	60 mg/kg intraperitoneally for 7 days; 15, 30, and 60 µM	cholestatic liver injury	Activation of the signaling pathway, including Sirt1 and FXR	[21]
In vivo	Male Wistar rats	25 and 50 mg/kg for 2 weeks	hepatoprotective activity	Nuclear factor kappa B is subsequently suppressed after Nrf2 and PPAR activation	[22]
In vivo and in vitro	Male Sprague Dawley rats and c57bl/6 mice and LO2 cells, HEK293T cells	30, 60, and 120 mg/kg in rats and 40 mg/kg in mice intraperitoneally for 5 days; 30 μM	reduction in acute liver injury	PXR-mediated inhibition of autophagy degradation	[23]
In vivo	Wistar rats	50 and 100 mg/kg, p.o.	hepatoprotective activity	Inhibit oxidative stress and inflammation via activating Nrf2 signaling	[24]
In vitro	Human CRC cell lines (LoVo, SW480, and SW620)	12.5, 25, 50, and 100 µM	antitumor effects against colorectal cancer	p-PI3K, p-AKT, p-STAT3, and p-NF-κB p65 protein levels were reduced	[25]
In vitro	Gastric cancer tissues and cell lines	50, 100, 150, and 100 µM	suppressed gastric tumorigenesis	Potentiating miR-149-3p-Wnt-1 signaling	[26]
In vitro	SGC-7901cells	20, 40, and 60 µM	prevention of gastric cancer metastasis	Prevents invasion and migration via the ROS/PKC-α/ERK signaling pathway	[27]
In vitro	Metastatic prostate cancer cell line LNCaP, DU-145, and HUVEC cells	100 and 200 µM	anti-inflammatory activity on prostate cancer cells	Matrix metallopeptidase-9, NF-κB, and vascular endothelial growth factor (VEGF) expression were all downregulated, whereas NSAID-activated gene-1 expression was elevated	[28]
In vitro	LNCaP human prostate cancer cells	0, 2.5, 5, and 10 μg/mL	human prostate cancer	Suppressed the expression of androgen target genes (TMPRSS2, prostate-specific antigen)	[29]
In vitro	Breast cancer cell	12.5, 25, 50, and 100 μM	inhibits the invasion and metastasis of breast cancer	Reducing p38 MAPK-AP1 signaling axis	[19]
In vitro	Human breast cancer cells, MCF-7	25, 50, 100, and 200 μM	antitumor properties	Caspase activation and modulation of Akt/forkhead box O3a (FOXO3a) pathway	[30]
In vitro	Human ovarian cancer a2780 cells	50 μM	induces apoptosis in ovarian cancer	A2780 cells expressed more Fas and FasL on their cell surfaces	[31]
In vitro	Non-small cell lung cancer (NSCLC) cells A549 and NCI-H460	80, 160, and 320 µM	inhibits non-small cell lung cancer	Inhibit extracellular signal-regulated kinase (ERK)1/2 and cyclic adenosine monophosphate response element-binding protein (CREB)	[32]
In vitro	A549 lung cancer cells	10, 20, 30, 40, and 50 μM	treatment of lung cancer	Induced apoptosis and G2/M cell cycle arrest	[33]
In vivo and in vitro	Xenograft nude mouse and HepG2 cells	20, 40, or 80 mg/kg; 5 and 10 μM	antihepatocarcinogenesis	Inhibition of IL-1β-induced activation of the IL-1R1/IκB/IKK/NF-κB signaling pathway	[34]
In vitro	Human epithelial ovarian carcinoma cells	15 and 25 µM	inhibition of epithelial ovarian adenocarcinoma	Hsp90 inhibition-induced apoptosis and activation of caspase-8	[35]
In vivo and in vitro	Immunocompetent C57BL/6 mice and mouse hepatoma cell line Hepa1-6	50 mg/kg, once daily (in vivo) for 3 weeks; 20 µg/mL for 3 days (in vitro)	protective role in hepatocellular carcinoma	Reducing T cell apoptosis and regulatory T (Treg) cell expression	[36]
in vitro	ΔKPQ Nav1.5 channels	1, 30, and 100 μmol/L	antiarrhythmic agent	Induces a tonic block of Ina	[37]
In vivo and in Vitro	C57BL/J6 mice	100 mg/kg for 10 days	inhibition of ischemic stroke	Antioxidant and significant decrease in lipid peroxidations	[38]
In vivo	Subcutaneous injection of ISO (85 mg/kg/day) in mice	50 and 100 mg/kg, gavage	cardioprotective effects on acute myocardial infarction	Inhibited PI3K/Akt signaling; reduced cell contractility and Ca^2+^ concentration	[39]

#### 3.1.2. Colorectal Cancer

The World Health Organization ranks colorectal cancer as the third most common form of the disease overall and as the fourth-greatest cause of death from cancer. The development of oncogene mutations, the deactivation of tumor suppressors, and numerous signal transduction pathways are just a few of the processes contributing to colorectal cancer. These processes also result in genetic abnormalities, dysregulated apoptosis, rising invasiveness, and morphological advancement. The function of 18βGA in colorectal cancer cells was explored by Wang et al. (2017). This research employed the human CRC cell lines LoVo, SW480, and SW620 and a normal human colon mucosal epithelial cell line (NCM460). Erk, p38, and JNK are three important mitogen-activated protein kinase (MAPK) families strongly related to cell proliferation, migration, and invasion in colorectal malignancies. PI3K/AKT signaling pathway is activated in cancer cells (SW480, LoVo, and SW620), enhancing cell survival, outgrowth, and migration. Further, 18βGA inhibited phosphorylation of phosphatidylinositol 3-kinase (PI3K), Akt, protein kinase B (AKT), signal transducer and activator of transcription 3 (STAT3), c-Jun N-terminal kinases (JNK), p38, and nuclear factor kappa B (NF-κB) p65 protein levels as well as cell migration, invasion, and wound healing [25] suggested that 18βGA might be potentially helpful in treating colorectal cancer patients (Table 1). 

#### 3.1.3. Pituitary Adenomas 

Further, adenomas of the pituitary gland, the most frequent form of all intracranial neoplasms, can elicit mass effects and hormone aberrant production symptoms. Pituitary adenomas cause cognitive abnormalities in most patients, especially memory and executive function [40]. In this study, researchers enrolled 647 individuals with pituitary adenoma, 135 of whom opted out or were disqualified from the study. In all, 512 individuals with pituitary adenoma finished the research, 268 of whom received 18βGA and 244 of whom received a placebo. Wang et al. (2018) exhibited that 18βGA has potential as a new chemotherapeutic for treating pituitary adenoma. The size of the tumor, the course of the disease, and hormone discharge tests were monitored in the study. After one month of surgery, orientation, communication, memorization, practicing, and analytical thinking, scores increased considerably in the 18βGA-treated group. However, 18βGA did not affect patient survival during the five-year follow-up but dramatically reduced the recurrence rate [40].

#### 3.1.4. Gastric Cancer

Further, the third-greatest cause of cancer-related death has been claimed tp be due to gastric cancer. Cai et al. (2018) explored the antimetastasis effect of 18βGA and the underlying mechanism of action in gastric cancer. They discovered that 18βGA suppresses invasion and metastasis in gastric cancer cells (SGC-7901 cells) through the ROS/protein kinase C-alpha (PKCα)/ERK signaling pathway, suggesting that it could be used as a chemopreventive drug to prevent gastric cancer metastases [41]. Cao et al. (2016) also assessed the effectiveness of 18βGA in gastric cancer. They reported that 18βGA reduced the genesis and progression of gastric cancer by improving inflammatory events, as it downregulates cyclooxygenase-2 (COX-2) expression and Wnt-1 expression and upregulates tumor suppressor miR-149-3p. Consequently, 18βGA may offer therapeutic promise in preventing and treating gastric cancer [26]. Numerous epidemiological studies have linked Helicobacter pylori (H. pylori) to stomach cancer. In addition, 18βGA has been reported to decrease the expression of inflammation-related genes in the antrum and corpus of gerbils with H. pylori-infected gastritis [27] (Table 1). 

#### 3.1.5. Prostate Cancer 

As the worldwide population of males over age 50 grows, the number of people diagnosed with prostate cancer continues to rise. It has been suggested that chronic inflammation, brought on by genetic and environmental factors, may play a crucial role in the development of cancer [42]. Shetty et al. (2011), revealed that 18βGA induced cell death in androgen-independent metastatic prostate cancer cells (DU-145). Prostate cancer cells preserved with 100 µM 18βGA for 48 h resulted in a 34% death of DU-145 cells. According to the findings, 18βGA suppressed the expression of inflammatory mediators’ high mobility group box protein 1, IL-6, and IL-8, which suggested treating prostate cancer [28]. 

In addition, Sun et al. (2020) found that the androgen receptor is critical in prostate pathology and tumor progression as an amplification target. 18βGA repressed androgenic and survival responses in prostate cancer cells by inducing tumor-suppressive miR-488 and transcriptional downregulation of AR via modulating E2F transcription factor 3 (E2F3)α and serum response factor (SRF) activity on the androgen receptor promoter [29]. Therefore, 18βGA could be a promising treatment option for men with prostate cancer.

#### 3.1.6. Ovarian Cancer 

The fatal gynecological disease in women is ovarian cancer. Despite improved prognosis for most solid tumors, the prognosis for epithelial ovarian cancer has remained stable in the previous 30 years, with only one crucial novel treatment introduced [43]. Furthermore, Haghshenas and coworkers (2014) stated that Fas binding to FasL causes receptor oligomerization and the development of a death-receptor signaling complex, which activates a sequence of caspase enzymes culminating in cell death. Tumor cells that express FasL destroy immune cells with a high concentration of Fas on their surface. On the membranes of human ovarian cancer cells (A2780), It has been proven that 18βGA increases the expression of FasL and Fas in cells [31]. 18βGA induces apoptosis in A2780 cells, suggesting that GA-induced apoptosis is at least partly triggered by Fas/FasL interaction, since GA treatment increases Fas and FasL expression on the cell surface of A2780 cells.

Furthermore, cancer cells have employed the molecular chaperone heat shock protein 90 (HSP90) to stimulate the action of various oncoproteins. It has been suggested that cancer cells are ‘addicted’ to HSP90 [44]. Heat, hypoxia, dietary deficiency, and oxidative injury activate heat-shock proteins. Allowing tumor cells to continue translating proteins and proliferating promotes survival, growth, and metastasis. Heat-shock proteins are typically overexpressed in human solid tumors and blood cancer. HSP90, a viable target for cancer therapy, causes oncogenic client protein activation important in signaling pathways, tumor growth and survivability, and apoptosis. Yang et al. (2012) discovered that increasing caspases 8, 9, and 3 activations might enhance the apoptotic impact of the HSP90 modulator on human epithelial ovarian tumor cell lines [35], indicating that 18βGA could be used in the handling of ovarian epithelial adenocarcinoma (Table 1).

#### 3.1.7. Lung Cancer 

Cancer deaths in the United States are mostly due to lung cancer, accounting for almost 85 percent of instances. The poor reactivity of this cancer to therapy, which has a 5-year survival rate that is very low (17.4 percent) and a high propensity to proceed to metastatic illness, is of particular concern [45]. Interestingly, in non-small cell lung cancer cells A549 and NCI-H460, Huang et al. (2014) reported the putative anticancer impact of 18βGA. 18βGA inhibited the ERK/CREB pathway by impeding TxAS, resulting in decreased cell propagation in non-small cell lung cancer [32] (Table 1). 

#### 3.1.8. Liver Cancer 

Liver cancer is predicted to be a major public health problem around the globe by 2025, with a predicted incidence of over one million cases. Ninety percent of all liver cancer cases are caused by hepatocellular carcinoma (HCC), the most frequent cancer. Infection with the hepatitis B and C viruses is the most prevalent trigger of hepatocellular carcinoma; however, nonalcoholic steatohepatitis linked with metabolic syndrome or diabetes mellitus is also a risk factor [46]. Surgical excision and liver transplantation are two alternatives for treatment. By blocking the actions of tumor-associated macrophages on tumor cells, a compound combining 18βGA and tetramethylpyrazine (TOGA) produced antihepatocarcinogenesis. The effect was attributed to inhibiting HepG2 cell invasion and migration produced by tumor-associated macrophages and activating the IL-1R1/IκB/NF-κB pathway in HepG2 cells. This study suggested that TOGA could be a viable 18βGA-modified medication for hepatocellular cancer treatment [34]. Additionally, hepatic stellate cells are immunosuppressive and play a role in the onset and progression of hepatocellular carcinoma. As a result, activated hepatic stellate cells could be a good target for hepatocellular cancer treatment [47]. Furthermore, by reducing FoxP3+ cell expression, T cell death and raising T cells in the spleen, 18βGA plays a significant function as an antitumor agent, providing solid evidence for 18βGA to be a promising contender as an anticancer treatment [36] (Table 1). 18βGA can halt the beginning and spread of cancer by focusing on molecular indicators of inflammation. These investigations uncovered the molecular processes by which 18βGA caused cancer cells to die, establishing its effectiveness as a powerful anticancer drug.

### 3.2. Antiarrhythmic Activity

The importance of the transient sodium current (INa,L) in arrhythmogenesis and its role in modulating cardiac action potential repolarization has received more attention [48]. In a concentration-dependent tonic way, 18βGA inhibited the INa,L produced by deletion of lysine, proline, and glutamine (ΔKPQ) Nav1.5 networks. In atrial cardiac cells, the higher inhibition of 18βGA on INa,L produced by anemone toxin II (ATX-II) was also seen. This research suggested that 18βGA has a lot of potential as a novel antiarrhythmic drug, especially for INa,L-related arrhythmias and myocardial ischemia [48] (Table 1).

### 3.3. Cerebral Ischemia

Stroke is the second major cause of mortality and disability. The majority of strokes are caused by an ischemic stroke due to arterial blockage [49]. When the blood movement to the whole brain or a significant brain region is cut off, tissue is deprived of oxygen and glucose, leading to tissue damage. Many mechanisms, including mitochondrial dysregulation, increased oxidative stress, blood–brain barrier disruption, brain inflammation, and increased neuronal death, are potential causes. Tissue plasminogen activator is the only medication now available for the acute therapy of stroke (t-PA). In different in vitro and in vivo experiments, plants with 18βGA exhibited neuroprotective effects. Oztanir et al. (2014) exhibited that 18βGA protected the brain against oxidative and histological damage induced by global ischemia/reperfusion. The study concluded that 18βGA could be a safe alternative treatment for ischemic stroke in people [38] (Table 1).

### 3.4. Hepatoprotective Activity

Methotrexate-induced hepatotoxicity is well known and causes an increase in liver marker enzymes and bilirubin and a decrease in albumin levels. Serum proinflammatory cytokines, malondialdehyde (MDA), and nitric oxide were considerably enhanced in methotrexate-induced hepatotoxicity [50]. A study by Mahmoud et al. (2017) reported that 18βGA showed its protective role by downregulating nuclear factor erythroid 2-related factor 2 (Nrf2), heme oxygenase-1, and peroxisome proliferator-activated receptors (PPAR)γ [20]. 

Further, hepatobiliary disease cholestasis has a variety of etiologies. Clinically diagnosed cholestatic liver disorders include primary biliary cholangitis and primary sclerosing cholangitis. Unfortunately, there are still limitations to how well therapeutic medications can cure hepatic cholestasis. Farnesoid X Receptor (FXR) agonist and secondary bile acid ursodeoxycholic acid are now the most widely used treatment methods for primary biliary cholangitis. Only a tiny percentage of patients, however, exhibit any positive response. To establish a therapy plan and perhaps find a medication candidate that can treat hepatic cholestasis, it is crucial to have a deeper knowledge of the molecular pathways behind the pathogenesis of cholestasis [51,52]. Wu et al., 2018, found that boosting the expression of the nuclear factors sirtuin 1 (Sirt1), Nrf2, and efflux transporter genes’ multidrug-resistance-associated protein 2 (Mrp2), bile salt export pump (Bsep) protects against cholestatic liver damage. 18βGA treatment-controlled cholestasis activates the Sirt1/farnesoid X receptor (FXR) and Sirt1/Nrf2 signaling pathways [21] (Table 1). Activating Sirt1, FXR, and Nrf2 reduces oxidative stress, inflammation, and programmed cell death by interacting with several intracellular signaling pathways. This activation also restores the homeostatic control of bile acid metabolism. Additional research is needed to fully comprehend the impacts and pleiotropic targets that 18βGA has attained as well as its enhanced value and use.

Another investigation identified cyclophosphamide-induced hepatotoxicity by increasing liver toxic markers. By activating antioxidant defense and reducing reactive oxygen species (ROS) production, 18βGA seemed to protect against cyclophosphamide-induced inflammation and oxidative stress. This was attributed to the coactivation of Nrf2 and PPARγ and subsequent control of NF-κB [22].

Furthermore, acute liver failure is a potential cause of death. Activation of the pregnane X receptor (PXR) has been reported by in vitro and in vivo studies to decrease acute liver damage. Wu et al. (2021) revealed that 18βGA prevented autophagosome-lysosome fusion, lowered cell death, and relieved liver damage via activation of PXR [23]. 

In addition, 2-acetylaminofluorene (2-AAF) is a recognized liver tumorigenic compound that causes hepatotoxicity and inflammatory stress. 2-AAF-induced lipid peroxidation, serum transaminase activities, phase II detoxifying enzyme activity, and glutathione levels have been reduced when 18βGA was used. The expressions of cyclooxygenase-2, iNOS, and NF-κB were lowered considerably after treatment with 18βGA. This research suggested that reducing oxidative stress, inflammation, and hyperproliferation could have hepatoprotective effects [53]. Yang et al. (2017) also reported that the hepatoprotective effect of low-dose 18βGA (50 mg/kg) was more significant than a higher dose of 100 mg/kg with triptolide-induced hepatotoxicity in rats [24] (Table 1). In a recent study, 18βGA also strengthened the Nrf2-mediated antioxidant mechanism, hence protecting the liver from damage brought on by retrorsine. To promote the nucleus accumulation of Nrf2 and activate its downstream targets, GA boosted the phosphorylation of PI3K/AKT and elevated glycogen synthase kinase 3 beta inhibitory phosphorylation at serine 9 [54]. Based on the various above investigations, 18βGA should be explored more for various liver disorders at the molecular level.

### 3.5. Thrombocytopenia

Immune thrombocytopenia (ITP) is an autoimmune bleeding condition. In ITP patients, a loss of peripheral immunological tolerance causes autoreactive T cells to multiply and produce autoantibodies against platelet cell surface antigens. HMGB1, a nonhistone nuclear protein, functions as a DNA chaperone and a damage-associated molecular pattern molecule outside the cell (DAMP). The pharmacological inhibitor of HMGB1 cytokine activity, 18-glycyrrhetinic acid (18-GA), is a new compound. PBMCs from ITP patients and healthy controls were isolated and cultured with DMSO or various dosages of 18-GA (12.5–100 μM) to see whether 18-GA might help the production and function of Tregs in ITP. By using flow cytometric analysis on PBMCs from ITP patients and healthy controls, the authors discovered a dose-dependent increase in the number of Tregs. In immunological thrombocytopenia, the authors discovered that 18-GA plays a regulatory function in maintaining CD4+ T cell homeostasis. By lowering the expression of HMGB1, 18-GA could diminish proinflammatory Th1, Th17, and Th22 cells while increasing the number and activity of Tregs. As a result, 18-GA might be a viable treatment option for ITP patients [55].

### 3.6. Anti-Inflammatory Activity

Inflammatory responses are mediated by the transcription factor NF-κB, which controls the expression of cytokines, intercellular adhesion molecule 1 (ICAM-1), different transcription factors, angiotensin-II, iNOS, and COX-2. 18βGA decreased cytokinin and stress response gene expression by inhibiting NF-κB nuclear translocation and exhibited anti-inflammatory activity [56] (Table 1). In a recent study by Zhang et al. (2022), 18β-glycyrrhetinic acid monoglucuronide reduced lung inflammation and fibrosis by single-walled carbon nanotubes. Days 3 through 28 following intratracheal injection of single-walled carbon nanotubes saw a rise in proinflammatory cytokines levels in bronchoalveolar lavage fluid and collagen deposition on Day 28. The treatment with 18β-glycyrrhetinic acid monoglucuronide significantly reduced the amount of pulmonary fibrosis brought on by single-walled carbon nanotubes, reduced the inflammation and collagen deposition it caused, and inhibited activation of the PI3K/AKT/NF-B signaling pathway in the lungs.

Consequently, 18β-glycyrrhetinic acid monoglucuronide may potentially cure pulmonary fibrosis [57]. In a recent study, Liu et al. (2022) reported that by preventing NF-κB phosphorylation and boosting the Nrf2/HO-1 pathway, 18βGA protected against OVA-induced allergic inflammation of the airways and may be used as a therapy for this condition [58]. The ability of 18βGA to control proinflammatory cytokines and inhibit the formation and progression of immune-inflammatory illnesses was shown in several experiments to be responsible for the therapeutic impact of 18βGA in inflammatory situations.

### 3.7. Antiasthmatic Activity

Bronchial asthma is a kind of chronic bronchitis characterized by incompletely reversible airway limitation. The main symptoms are shortness of breath and wheezing, abnormal bronchial smooth muscle proliferation, massive inflammatory cell infiltration, and inflammatory factor secretion, important pathological characteristics in the local airway [59]. The involvement of 18βGA in controlling inflammatory responses in asthma has received increasing attention. The major metabolite of licorice, 18βGA, is a mainstay of Chinese medicine. 18βGA has been observed to lower airway hyperresponsiveness, inflammatory cell accumulation, and elevation of peroxisome proliferator-activated receptor-γ mRNA expression. The action of 18βGA was established by overexpression of the forkhead box p3 gene and underexpression of STAT6 (signal transducer and activator of transcription 6). 18βGA was identified as a unique healing constituent for treating hypersensitive asthma in this investigation [60]. Further studies on 18βGA in asthma should be explored to find better treatment options in hypersensitive asthma.

Additionally, Zhang et al. (2017), also evaluated the role of 18βGA on male SD guinea pig bronchial smooth muscle cells and elucidated the mechanism of its action. The study reported that 18βGA inhibits the phosphorylation of extracellular signal-regulated protein kinase (ERK) 1/2 to promote apoptosis, impedes the discharge of inflammatory cytokines in the bronchial smooth muscle airway, and may help regulate the expression of inflammatory factors in asthma [61]. 

Furthermore, the recruitment and stimulation of inflammatory cells cause airway inflammation. Monocyte chemo-attractant protein 1 (MCP-1) and interleukin (IL)-8 are multifunctional inflammatory cytokines induced by Aspergillus protease. MCP-1 activates neutrophil recruitment by stimulating migrating macrophages and monocytes. MCP-1 and IL-8 production by Aspergillus protease is reportedly inhibited by 18βGA. 18βGA also reduced in vivo epithelial inflammation caused by Aspergillus protease activation. The mitochondrial uncoupling proteins-2 (UCP-2) expression regulation is required for these inhibitory actions [62]. 18βGA has been suggested as a prospective healing drug for reducing lung inflammatory reactions (Table 1). 

### 3.8. Nephroprotective Effects

Anticancer medications frequently induce harmful effects in patients, causing current cancer therapy to be discontinued. Renal impairment, tubular inflammation, hyaline casts, and tubular atrophy are recognized side effects of anticancer drugs such as methotrexate and cisplatin, which increase blood urea nitrogen, creatinine, and lactate dehydrogenase levels [63]. Methotrexate (dihydrofolate reductase inhibitor) has been used to treat human cancer and autoimmune illnesses. In clinical medicine, natural materials are frequently used as adjuvants. 18βGA has been demonstrated to have nephroprotective effects in the kidney by reversing tubular damage via Nrf2 and downregulating NF-κB. The anti-inflammatory activity of 18βGA in methotrexate-induced rats may be due to the upregulation of Nrf2 expression. Nrf2/ARE is one of the systems that controls inflammation [64] (Table 2).

**Table 2:** Nephroprotective, antidiabetic, antiasthmatic, and management of pulmonary arterial hypertension and mechanisms of 18β-glycyrrhetinic acid in both in vitro and in vivo.

Assay	Model	Dose/Concentration of 18β-Glycyrrhetinic Acid	Effect	Mechanisms	Reference
In vivo	Cisplatin-induced renal injury in male BALB/c mice	25, 50, and 100 mg/kg	nephroprotective effects	Overexpression of Nrf2 and reduced expression of NF-κB in the kidney	[64]
In vitro and in vivo	HK-2 and mTEC cells lines; cisplatin-induced AKI in C57BL/6 mice	2.5, 5, 10, 20, 30 μM (in vitro); 50, 100, and 200 mg/kg (in vivo)	nephroprotective effects	Enhancing BMP-7 epigenetically through targeting HDAC2	[65]
In vivo	Rats	50 and 100 mg/kg, oral gavage for 7 days	nephroprotective effects	Upregulating the Nrf2/ARE/heme oxygenase 1 (HO-1) pathway and endogenous antioxidants	[66]
In vivo	Single-dose of 50 mg/kg streptozotocin (STZ) intraperitoneally in rats and 20 mg/kg of acrylamide	50 mg/kg, orally	inhibit reactive oxygen species generation	Decrease serum glucose, cholesterol, creatinine, IL-1β, IL-6, TNF-α	[67]
In vitro	High glucose (HG)-induced THP-1 cells	12.5, 25, and 50 µM	implication to diabetes mellitus	Decrease expressions of ROS, p47s, and iNOS and increase UCP2 levels, promotinga soluble form of RAGE (sRAGE) secretion	[68]
In vivo	Streptozotocin-diabetic rats	50, 100, or 200 mg/kg, oral	antihyperglycemic effect	Increase plasma insulin and lower glycosylated hemoglobin	[69,70]
In vivo and in vitro	Sprague Dawley rats and human pulmonary arterial smooth muscle cells	25, 50, and 100 mg/kg (in vivo); 20, 40, 80, and 160 μM	antiangiogenic effect on pulmonary arterial hypertension	Expression of Rho A, ROCK1, and ROCK2 was decreased, and ROCK activity was inhibited	[71]
In vivo	BALB/c mouse model of allergic asthma	2 and 20 mg/kg, oral	antiasthmatic activity	Inhibition of the RORγt, STAT6, and GATA-3 pathways, as well as activation of the Foxp3 transcription pathway	[60]
In vitro	Male SD guinea pigs’ bronchial smooth muscle cells	50 ng/mL	antiasthmatic activity	Inhibiting the phosphorylation of ERK1/2	[57]
In vitro	Mouse BALB/c macrophage cell line (RAW264.7)	1 and 5 μM	antiasthmatic activity	Blocking inflammation via PI3K/Akt/GSK3β signaling and dissociating a glucocorticoid receptor-HSP90 complex	[72]
In vitro and in vivo	Human bronchial epithelial cell line BEAS2B; C57BL/6 mice	5, 10, 15, 20, and 25 μM; 50 mg/kg, oral	inhibition of airway; inflammation	Suppression of the mitochondrial ROS/MAPK axis	[62]
In vivo	Neonatal rats with hyperoxia exposure	50 or 100 mg/kg, intragastrically	protected neonatal rats with hyperoxia exposure	Reduced ROS and prevented the activation of NF-κB and the NLRP3 inflammasome	[73]

Further, 18βGA decreased cisplatin-induced higher levels of kidney injury molecule-1 in the cell lines HK-2 and mTEC. Ma et al. (2016) discovered new evidence for 18βGA’s preventive effect in C57BL/6 mice with acute renal injury caused by cisplatin. By raising BMP-7 and HDAC2 levels in renal tubular epithelial cells, 18βGA was able to minimize apoptosis in these cells and protect against cisplatin-induced acute nephrotoxicity. Thus, 18βGA was suggested as a therapy for progressive acute kidney injury [65]. 

Abd El-Twab et al. (2016) also looked at 18βGA’s ability to protect against methotrexate-induced nephrotoxicity. 18βGA improved kidney function indicators, renal lipid peroxidation, reactive nitrogen species, and antioxidant defenses. Moreover, supplementation with 18βGA exhibited nephroprotective effects by upregulating Nrf2 and heme oxygenase-1 mRNA in kidneys with methotrexate-induced nephrotoxicity [66] (Table 2). Caglayan et al. (2022) recently examined 18βGA’s impact on bisphenol-induced neurotoxicity. Wistar albino rats received 50, 100, and 200 mg/kg 18βGA. Bisphenol poisoning increased MDA and decreased GSH, superoxide dismutase, and catalase. BPA triggered apoptosis by upregulating caspase-3 and Bax and downregulating Bcl-2. Bisphenol increased PERK, IRE1, ATF-6, and GRP78 mRNA transcript levels, causing endoplasmic reticulum stress. Bisphenol also stimulated JAK1/STAT1 signaling. Cotreatment with 18βGA at 50 and 100 mg/kg significantly reduced oxidative brain damage, inflammation, apoptosis, ER stress, and JAK1/STAT1 signaling [74]. This research found that 18βGA might reduce bisphenol-related brain damage. These studies uncovered the molecular mechanisms through which 18βGA had a nephroprotective effect and may be useful in treating kidney diseases.

### 3.9. Antidiabetic Activity

Over the last three decades, there has been a worldwide increase in the occurrence of diabetes mellitus, and it is currently the ninth-greatest cause of death. Type 2 diabetes accounts for the vast majority of diabetes today, affecting 1 out of every 11 individuals worldwide. The worldwide type 2 diabetes pandemic has a substantial hotspot in Asia, with China and India as the top two epicenters [75]. Diabetes mellitus and its complications are treated globally with ancient therapeutic compositions based on plants and their potent phytoconstituents. These herbal medications have been shown to slow the progression of diabetes complications and change metabolic abnormalities [76]. Alanazi et al. (2021) recently revealed that 18βGA protects diabetic rats from acrylamide-induced cellular damage. Streptozotocin and acrylamide increased oxidative stress in the liver and kidneys, which was reversed by a 18βGA injection [67]. Another study discovered that 18βGA (100 mg/kg) has a putative antidiabetic effect comparable to glibenclamide [69] (Table 2).

Furthermore, 18βGA was shown to be a partial antagonist of hepatocyte nuclear factor 4 alpha (HNF4α) using HNF4-driven reporter luciferase tests. Serine 190 and arginine 235 of HNF4α are required for 18βGA to exert its antagonistic activity on HNF4α to be effective, according to virtual docking studies. By downregulating the expression of HNF4α target genes, 18βGA reduced gluconeogenesis and improved glucose intolerance and is suggested for antidiabetic use [77] (Table 2).

In an in vitro THP-1 cells study, 18βGA revealed its effects through the soluble receptor for advanced glycation end product release via transient receptor potential channel (TRPC). 18βGA blocked protein expression (TRPC3 and TRPC6), suppressed intracellular calcium availability, decreased oxidative stress and inducible nitric oxide synthase (iNOS), and increased uncoupling protein 2 levels induced by high glucose [68] (Table 2). As a consequence, 18βGA may be useful as a therapeutic agent in the management of diabetes mellitus.

### 3.10. Pulmonary Arterial Hypertension

Pulmonary arterial hypertension (PAH) is indicated by pulmonary arterial pressures above 25 (rest) and 30 (active) millimeters of mercury. PAH is manifested by pulmonary artery resistance and a rise in pressure in the pulmonary arteries, which impact the right ventricular enlargement and can lead to death [78]. Pulmonary artery smooth muscle cells proliferate, migrate, resist apoptosis, and produce PAH. Zhang et al., (2019) found that 18βGA decreased smooth muscle cell proliferation by modulating the RhoA/rho-associated coiled-coil kinase (ROCK) signaling pathway activity. Furthermore, 18βGA inhibited platelet-derived growth factor-induced alterations in p27kip1 and Bcl-2, suggesting that it could be used to treat pulmonary arterial hypertension [71] (Table 2). In another investigation, intragastric administration of 18βGA for 21 days also protected rats from monocrotaline (60 mg/kg)-induced PAH by impeding the expression levels of Nox2 and Nox4 enzymes [71] (Table 2). Furthermore, elevated blood pressure increases gap junctional channel activities in smooth muscle cells [79]. 18βGA was discovered to block cerebral arteriolar gap junctions between artery smooth muscle cells in a dose-dependent manner in an in vitro study. The Wistar rat’s IC50 for suppressing G(input) was 1.7 micromol/L, while the spontaneously hypertensive rat’s IC50 was 2.0 micromol/L.

### 3.11. Antileishmanial Effect

Leishmaniasis is a parasitic illness spread by vectors that affect 12 million people across the globe and accounts for 0.5 million new cases each year. Antileishmanial medications are now scarce, and the treatment regimen is extensive. However, these medications have severe side effects, and refractory cases are a concern. T lymphocytes, NK cells, and macrophage immunological impairment are linked to the condition [80]. An investigation by Gupta et al. (2015) in gene knockout studies has shown that toll-like receptors (TLR) signaling is critical in the immune response against Leishmania parasites. The antiparasitic effects of 18βGA were shown using TLR-2-dependent ubiquitin-dependent kinase of MKK and IKK (TAK1)/MKK-3/6 and p38NF-κB activation. 18βGA modulated the phosphorylation of p38 in macrophages and offers new possibilities for creating novel medications that are effective against intracellular parasites in the future [81]. In diseased bone marrow-derived macrophages, 18βGA reduced the severity and activity of three phosphatases and demonstrated antileishmanial activity via inhibiting the p38 and ERK pathways [82] (Table 3). Consequently, 18βGA is now a viable option for administering an antileishmanial treatment.

**Table 3:** Antileishmanial, antiviral, antibacterial, antifungal, and prevention of psoriasis and mechanisms of 18β-glycyrrhetinic acid in in vitro and in vivo.

Assay	Model	Dose/Concentration of 18β-Glycyrrhetinic acid	Effect	Mechanisms	Reference
In vitro	Murine macrophage cell line RAW 264.7	20 μM for 4 h	antileishmanial effect	Toll-like receptor-dependent canonical and noncanonical p38 activation	[81]
In vivo	L. donovani-infected BALB/c mice	10, 50 and 100 mg/kg i.p. for 3 times	antileishmanial effect	Inhibition of p38 and ERK pathway	[82]
In vitro	Rhesus rotavirus strain RRV was propagated in MA104 cell	25 μg/mL	antiviral activity against rotavirus replication	VP2, VP6, and NSP2 were reduced	[83,84]
In vitro	MA104 cells infected with rotavirus	1, 2, 4, and 8 μg/mL	inhibit cells infected with rotavirus SA11	Fas/FasL pathway	[23]
In vivo	Murine hepatitis virus (MHV) infection model	10, 100 and 1000 μg/mL	against hepatic inflammation injury in viral hepatitis disease	Inhibition of viral-induced HMGB1-TLR4 immunological regulation axis	[85]
In vitro	MRSA strainsaeR and hla	600 μg	bactericidal to MRSA	Decreasing the expression of saeR, hla, mecA, and sbi	[86]
In vitro	Streptococcus mutans and Streptococcus sobrinus	32, 64, 128 and 256 μg/mL	antibacterial agent		[87]
In vivo	Mongolian gerbils	0.1% concentration	attenuated H. pylori-infected gastritis	Decrease expression levels of TNF-α, IL-1β, COX-2, and iNOS	[27]
In vivo	BALB/c mice	500 μg/mL	antifungal against Candidal infection	Induced immunological adjuvant activity of Th1 against Candida albicans	[88]
In vivo	Male BALB/c mice	60 and 120 mg/kg for 7 days	prevention of psoriasis	Suppression of mTOR/STAT3 signaling	[89]
In vitro and in vivo	Human HaCaT keratinocytes and C57BL6 mice	10, 20, 40, and 80 µM (in vitro); 50 mg/cm^2^ cream twice daily for 7 consecutive days (in vivo)	prevention of psoriasis	Inhibition of ROS-mediated PI3K-Akt signaling pathway	[90]

### 3.12. Antiviral Action

Rotavirus infections cause severe watery diarrhea in children under 5 years of age. Although rotavirus immunizations were widely available over a decade ago, rotavirus infections continue to be responsible for the deaths of over 2 lakh individuals per year, the vast majority of whom reside in underdeveloped countries [91]. In a study by Hardy et al., (2012), when 18βGA was introduced to infected cultures after virus adsorption, it reduced viral proteins VP2, VP6, and NSP2 by 99 percent, revealing antirotavirus activity [83] (Table 3). Furthermore, hepatitis caused by a virus is a severe public health concern and a prominent cause of worldwide demise. By suppressing high mobility group protein box 1 (HMGB1) cytokine activity and causing TLR4 gene deficit, 18βGA therapy reduced hepatic inflammatory damage in viral hepatitis. Hepatoprotective action in viral hepatitis happens due to the viral-provoked HMGB1-TLR4 immunological regulation axis. This research revealed a novel treatment method for acute viral hepatitis [85] (Table 3). 

### 3.13. Antibacterial Activity

A renewed focus on developing novel antibacterial compounds that could create more efficient alternative treatment approaches is necessary in light of the rapid development of bacterial resistance to the most commonly prescribed antibiotics. Most antibiotics have been derived from natural sources, and these products and their derivatives are rich sources of new compounds. The prevalence of antibiotic-resistant Staphylococcus aureus (Methicillin-resistant Staphylococcus aureus, or MRSA) strains is rising, which calls for innovative new therapies [92]. In a study by Krausse et al., the researchers compared the effectiveness of glycyrrhetinic acid monoglucuronide acetylated, glycyrrhetinic acid, and glycyrrhizic acid against 29 different strains of Helicobacter pylori. They discovered that glycyrrhetinic acid suppressed 79.3 percent of bacteria strains [93]. Long et al. (2013) demonstrated that 18βGA had bactericidal efficacy against MRSA by lowering the presence of MRSA’s primary virulence genes, saeR, mecA, and sbi [86]. Another study suggests that 18βGA is a capable natural drug for averting the onset and progression of periodontal disease [87] (Table 3).

In a recent investigation, 18βGA (7.8 mg/L) impaired bacterial cell-to-cell aggregation compared to controls, and significantly higher CFU counts accompanied a decrease in Staphylococcus aureus bacterial cell-to-cell aggregation at 18βGA concentrations below the MIC (i.e., 62.5 mg/L) [94]. 18βGA, in conjunction with other therapy regimens, might restore antimicrobial activity when administered alone, owing to antibiotic resistance. The physiological effects of 18βGA at sub-MIC doses may help limit future 18βGA resistance, minimizing antimicrobial resistance in *S. aureus*.

### 3.14. Antifungal Activity 

Candida spp. is the most prevalent cause of opportunistic mycoses throughout the globe. Candida yeasts are linked to various clinical symptoms, including superficial skin and mucosal surface infections and systemic and potentially life-threatening illnesses in healthy people. In BALB/c mice, 18βGA elicited a more robust immune reaction of Th1 than Th2 against Candida albicans surface mannan extract, indicating antifungal efficacy [88] (Table 3).

### 3.15. Antipsoriasis Effects

Psoriasis is a prolonged inflammatory immune-related skin condition regarded as hyperkeratosis, dermis, epidermis immune cell infiltration, and angiogenesis. Untreated psoriatic patients have an increased risk of developing cardio-metabolic disorders and malignancy [95]. Interestingly, 18βGA inhibited the expression of cytokines in the skin and raised the fraction of regulatory T cells (Tregs), which improved psoriatic lesions and harshness scores. Its anti-inflammatory and immunomodulatory properties were influential in treating psoriasis via suppressing STAT3 and mTOR signaling [89]. Another study discovered that 18βGA lowered viability and induced cell death in HaCaT keratinocytes. ROS formation of HaCaT keratinocytes and, consequently, the suppression of PI3K-AKT signaling were shown to be associated with apoptosis, suggesting that 18βGA might be put to use in the development of antipsoriatic drugs in the future [90] (Table 3).

### 3.16. Skin Diseases

UVB radiation is a primary etiological factor in developing skin cancer and aging. New ways to prevent and repair UVB damage are required to minimize sun-induced skin cancer. Inflammation caused by radiation is a major factor in radiation-induced tissue damage [96]. In exposed RAW264.7 macrophages, 18βGA administration reduced prostaglandin E2 (PGE2) production and p38MAPK phosphorylation and NF-κB activation. The authors of this study discovered that 18βGA inhibits oxidative stress, p38MAPK, and nuclear factor-kappa signal activation, which lowers proinflammatory cytokine release and possesses anti-inflammatory characteristics against radiation-induced skin injury [97].

### 3.17. Rheumatoid Arthritis

Rheumatoid arthritis is an inflammatory illness that causes joint inflammation, cartilage, and bone degradation, resulting in increased disability. Puchner et al. (2012) created transgenic mice using the human tumor necrosis factor (TNF) gene construct to induce arthritis with pannus development, cartilage breakdown, and bone erosion, identical to human rheumatoid arthritis. Unfortunately, however, the authors did not receive any positive effects of 18βGA in the TNF-α triggered mouse model of rheumatoid arthritis. The authors stated that failure in the study might be due to various medication administration methods or pure phytochemicals, which are inadequate compared to licorice extract [98].

### 3.18. Antiosteoporotic Activity 

Osteoporosis is the most frequent osteoclastogenesis-related illness. The most frequent type of osteoporosis is postmenopausal osteoporosis, caused by a lack of estrogen. Macrophage colony-stimulating factor (M-CSF) and receptor activator of nuclear factor-kappa-Β ligand (RANKL) are the two critical factors in osteoclastogenesis [99]. Microstructural damage causes increased bone brittleness and the possibility of fracture. Chen et al. (2018) discovered that 18βGA inhibited osteoclastogenesis, actin formation, and osteoclast functions by preventing RANKL-induced NF-κB/ MAPK signal transduction pathways at an early stage. In ovariectomized mice, 18βGA protected against ovariectomy-induced bone loss by increasing the mineral apposition rate [100]. Furthermore, this research suggested that for osteoclast-related degenerative bone illnesses, such as postmenopausal osteoporosis, 18βGA may be a promising treatment option. In a recent study, Chen et al. (2021) discovered that 18βGA-reduced IL-1β provoked an inflammatory response in mouse chondrocytes and slowed osteoarthritis development via activating the Nrf2 gene [101] (Table 4). Therefore, 18βGA seems to be a new agent that might be further investigated as a therapy for osteoporosis.

**Table 4:** Management of postmenopausal osteoporosis, osteoarthritis, antipsychotic-induced hyperprolactinemia, multiple sclerosis, and inflammation and mechanisms of 18β-glycyrrhetinic acid in in vitro and in vivo.

Assay	Model	Dose/Concentration of 18β-Glycyrrhetinic Acid	Effect	Mechanisms	Reference
In vivo and in vitro	Bone marrow monocytes (BMMs), RAW264.7 cells, and C57BL/6 female mice	6.9535, 13.905, and 27.81 μg/mL (in vitro); 50 mg/kg, intraperitoneally	inhibition of postmenopausal osteoporosis	Inhibited osteoclastogenesis by blocking RANKL-mediated RANK-TRAF6 interactions and NF-κB and MAPK signaling pathways	[100]
In vivo and in vitro	Chondrocytes and mouse model	50 mg/kg	inhibition of osteoarthritis	NF-κB activation caused by IL-1 was prevented by activating the Nrf2/HO-1 pathway	[101]
In vitro and in vivo	Collagen-induced arthritis mouse model	100 and 200 μM (In Vitro) 45 mg/kg, p.o. (In Vivo)	inhibit rheumatoid arthritis	Inhibition of MAPK/NF-κB and promotion of FOXO3 signaling	[102]
In vitro	Sprague Dawley rats mesenteric artery preparation	30, 40 μM, 45 min	gap junction blocker	Depolarize the mitochondrial membrane potential by inhibiting IP3-mediated Ca^2+^ release	[103]
In vivo	Rats	5, 10, and 20 mg/kg, intragastrically	reduces antipsychotic-induced hyperprolactinemia	Inhibited prolactin hyperactivity and also modulated the expression of 5-HT1A and 5-HT2A receptors	[104]
In vivo and in vitro	MOG35–55-immunized mice	75 mg/kg i.p. daily from day 7 or day 11 (In vivo) and 25 μM and 50 μM (In vitro)	multiple sclerosis	Inhibition of microglia activation and promotion of remyelination through suppression of MAPK signal pathway	[105]
In vitro	Irradiated RAW264.7 macrophages	10 μg/mL	anti-inflammatory actions against radiation-induced skin damage	Inhibition of NADPH oxidase/ROS/p38MAPK and NF-κB pathways	[97]
In vitro	RAW 264.7 cells	20 μM	anti-inflammatory activity	Inhibited the gene expressions of COX-2, iNOS, and NF-κB	[56]
In vitro	Kv1.3 channels in Jurkat T cells	10–100 µM	anti-inflammatory and immunomodulation effects	Blocked Kv1.3 potassium channels and T cell activation in human Jurkat T cells	[106]
In vitro	Everted rat gut sac model	1 mM and 100 μM	increase bioavailability	Inhibition of efflux transport mediated by intestinal P-gp	[107]

### 3.19. Antiepileptic Effects

Gap junctions are intercellular channels that allow ions to move bidirectionally into the cytoplasm of neighboring cells. The formation of highly synchronized electrical activity is aided by the electrical connection that is mediated by gap junctions. Convulsive episodes are distinguished by their hypersynchronous neural activity. The onset and maintenance of seizures are linked to increased gap junctional transmission. Because of their potential to interfere with cell communication through gap junctions, several chemical compounds are categorized as gap junction blockers. Because of its gap junction blocker feature, 18βGA has a potential future in searching for novel pharmacological solutions to treat epilepsy [103,108] (Table 4).

### 3.20. Antipsychotic-Induced Hyperprolactinemia

The most common cause of nonphysiological hyperprolactinemia is drug-induced hyperprolactinemia. Hyperprolactinemia appears to directly influence pancreatic beta cells, making it a risk factor for obesity-related metabolic syndrome [109]. In a rodent model of hyperprolactinemia instigated by repetitive metoclopramide injections, Wang et al., 2016, found that 18βGA decreased increased prolactin levels and growth hormones and balanced many sex hormones. With in vivo and in vitro models, 18βGA influenced the expression of 5-hydroxytryptamine (5-HT)1A and 5-HT2A receptors. These findings suggest that 18βGA is beneficial in reducing prolactin hyperactivity caused by dopamine D2 receptor blockade. This study adds to the evidence supporting the use of 18βGA as a supplement in treating hyperprolactinemia [104] (Table 4).

### 3.21. Multiple Sclerosis

Multiple sclerosis is a neuroinflammatory disease that may lead to progressive disability and the development of demyelinating lesions in the central nervous system, followed by neuro-axonal damage [110]. Microglia activate quickly in multiple sclerosis, producing massive levels of proinflammatory cytokines that mobilize additional immune cells, resulting in oligodendrocyte demise and demyelination. Furthermore, 18βGA offered a unique treatment potential because it inhibited the MAPK signal pathway, modulating microglial activity. 18βGA-modulated microglia inhibited T cell recruitment and relieved demyelination. Importantly, 18βGA boosted remyelination by increasing OPC proliferation, which could be attributable to 18βGA-modulated microglia increasing brain-derived neurotrophic factor (BDNF) in the central nervous system. Research suggested that microglia management might lead to a novel treatment strategy for multiple sclerosis and other neurological diseases [105,111] (Table 4). 

### 3.22. Pseudoaldosteronism

Excessive licorice consumption is known to cause pseudo-aldosteronism, a condition marked by peripheral edema, hypokalemia, and hypertension. Licorice is a traditional Chinese crude medication made from the roots of both *Glycyrrhiza glabra* and Glycyrrhiza ularensis. One of the most common adverse effects of licorice consumption is hypokalemia, also known as pseudoaldosteronism. The use of licorice is contraindicated in those who have high blood pressure as a result of licorice overuse.

In the distal nephron, cortisol is broken down into inactive cortisone by an enzyme called type 2 11-hydroxysteroid dehydrogenase (11HSD2), blocked by glycyrrhizin metabolites. The most common glycyrrhizin metabolite detected in people following licorice eating is 18β-glycyrrhetyl-3-O-sulfate, followed by glycyrrhetinic acid. In constipated individuals, glycyrrhizin is efficiently converted to glycyrrhetinic acid by intestinal bacteria, improving the bioavailability of glycyrrhizin metabolites. At the distal nephron, unbound metabolite fractions may reach 11βHSD2 under hypoalbuminemia circumstances. Pseudo-aldosteronism is caused by the compound 18β-glycyrrhetyl-3-O-sulfate [112].

Glycyrrhizic acid and ammonium glycyrrhizate and their metabolites have been shown to cause sodium and fluid retention, weight gain, suppression of the renin–angiotensin–aldosterone system, and high blood pressure. Both glycyrrhetinic and glycyrrhizic acids work together to prevent carbon tetrachloride from coming into contact with liver tissue. Glycyrrhizic acid has been revealed to be effective in treating chronic hepatitis by obstructing entry of the hepatitis A virus into hepatocytes, the target cells of the disease. Both glycyrrhetinic acid and glycyrrhizic acid have been shown to have anti-inflammatory effects in rodent models, namely rats and mice. In mice, the acute LD50 for glycyrrhetinic acid intraperitoneally was 308 mg/kg, whereas the oral LD50 was more than 610 mg/kg [113].

Further, in an in vitro test using shaved rabbit skin, glycyrrhetinic acid was found not to be irritating; nonetheless, the test found it moderately irritating. Glycyrrhetinic acid was shown to inhibit the carcinogenic effects of benzo[a]pyrene in mice and initiate and stimulate tumor growth by other agents. In mice, glycyrrhizic acid reduced the number of new tumors that developed but did not inhibit the growth of existing tumors. In a study that lasted for 96 weeks and used drinking water as the subject, rats that were given dosages of disodium glycyrrhizate ranging from 0 to 12.2 mg/kg day^−1^ were not observed to develop cancer. There was no evidence of either reproductive or developmental harm caused by glycyrrhizate salts in rodents. Sedation, hypnosis, coldness, and respiratory depression were seen in mice that had received intraperitoneal administration of 1250 mg/kg of glycyrrhetinic acid. In this experiment, rats were fed a powdered meal of up to 4 percent ammonium. In the tests that evaluated motor function, glycyrrhizate did not show any treatment-related effects. Even if there are holes in our knowledge of product use, the aggregate data on the types of products in which these chemicals are used and at what concentrations reveal a pattern of use. This is the case even though there are gaps in our understanding of product usage. Within this general pattern of use, an expert panel considers all the chemicals that fall under this category as risk-free [113].

### 3.23. Increase Bioavailability

18βGA is said to be a P-glycoprotein and multidrug-resistance protein inhibitor. As a result, 18βGA may work in concert with other elements in traditional Chinese medicine prescriptions during therapy by limiting these components’ efflux and increasing their effectiveness. Glycyrrhizin and licorice, for example, had a considerable effect on methotrexate pharmacokinetics in rats, which might be explained by the greater blood level of 18βGA [114]. The licorice aided in the absorption and concentration of peoniflorin in peonies. Some alkaloids’ AUC was raised by glycyrrhizic acid. 18βGA was also reported to increase intestinal absorption of paeoniflorin via P-glycoprotein (P-gp) inhibition [30].

## 4. Pharmacokinetics of Glycyrrhizin and Glycyrrhetinic Acid

The effect that three different kinds of active compounds found in traditional Chinese medicine (TCM) have on the pharmacokinetics of glycyrrhetinic acid (GA), which is a type of active component found in licorice, the TCM herb that is used frequently, was studied using a validated high-performance liquid chromatography technique. A very sensitive liquid chromatography–tandem mass spectrometry (LC-MS/MS) approach was validated and used for a human pharmacokinetic investigation to simultaneously detect glycyrrhizin and its active metabolite, glycyrrhetinic acid, from human plasma. Glycyrrhizin was found in measurable amounts in the peripheral blood of all participants. After the oral administration of glycyrrhizin, the concentrations of glycyrrhizin and glycyrrhetinic acid were measured over time. Following injection, glycyrrhizin was quickly absorbed, with first (0.5 h) and second (6 h) peaks and mean Cmax and Tmax of 24 ng/mL and 4.5 h, respectively. On the other hand, the 238 ng·h/mL of AUC0-t glycyrrhetinic acid was scarcely detectable 4 h after GL consumption and quickly increased after 6 h. Glycyrrhetinic acid had a Cmax of 200 ng/mL, eight times greater than glycyrrhizin. Glycyrrhetinic acid levels steadily dropped with time after attaining Tmax (10.3 h); however, glycyrrhetinic acid was detectable in some patients even after 48 h. Glycyrrhetinic acid has an AUC0-t of 3550.8 ng·h/mL. It is possible that the discrepancy in glycyrrhetinic acid plasma concentrations among the six participants was attributable to differences in intestinal flora [113].

## 5. Drugs in the Clinical Trial of 18βGA and Related Compounds

Licorice root has been shown to have several compounds with neuroprotective properties. Ravanfar et al. (2016) performed a randomized, double-blind, placebo-controlled experiment on 75 individuals with acute ischemic stroke. Patients were given capsules containing either 450 mg or 900 mg of licorice extract or a placebo for seven days. Patients ingested the capsules three times each day. The results of this study point to the potential utility of whole licorice extract in treating acute ischemic stroke patients’ neurologic symptoms [114]. Further, patients are being sought by Qilu Hospital of Shandong University in China to test the efficacy and safety of diammonium glycyrrhizinate enteric-coated capsules with high-dose dexamethasone for the treatment of persons with recently diagnosed primary immune thrombocytopenia. One group of participants was given diammonium glycyrrhizinate enteric-coated capsules orally at a dosage of 150mg tid for 3 months, in combination with dexamethasone (given orally at a dose of 40 mg QD for 4 days). The others was given a high dose of dexamethasone on their own. This study’s objective was to determine how efficient and safe diammonium glycyrrhizinate enteric-coated capsules combined with high-dose dexamethasone therapy are for treating ITP (NCT05023915). Furthermore, using a meta-analysis technique, Yen et al. (2014) investigated the effectiveness and safety of the combined therapy of magnesium isoglycyrrhizinate and nucleoside analogs (MGL + NA) in individuals with chronic hepatitis B. According to the findings, MGL combination therapy may improve liver function and boost the antiviral effectiveness of NA treatment in patients with chronic hepatitis B (NCT03349008). This supports 18βGA’s efficacy alone and in conjunction with common medications.

Moreover, glycyrrhetinic acid and dexamethasone are being tested in phase 4 clinical trials to treat immunological thrombocytopenia (ITP) that has just been identified. With 30 adult ITP patients, the researchers want to perform a parallel-group, single-center, randomized controlled trial. One group of participants is randomly allocated to undergo high-dose dexamethasone therapy with a placebo. In contrast, the other group is assigned to receive glycyrrhetinic acid treatment plus dexamethasone (given orally at 40 mg daily for 4 days, two cycles with a 10-day gap). Before and after therapy, the platelet count, hemorrhage, and other symptoms were assessed. Throughout the trial, adverse occurrences are documented to explore the efficacy and tolerability of glycyrrhetinic acid combined with high-dose dexamethasone treatment in ITP patients (ClinicalTrials.gov; NCT03998982). The current status of all clinical trials for 18β-on glycyrrhetinic acid are given in Table 5.

**Table 5:** Current status of clinical trials on glycyrrhetinic acids.

NCT Number	Title	Status	Conditions	Interventions	Outcome Measures	Sponsor/Collaborators	Phases	Enrollment	Completion Date
NCT03998982	Glycyrrhetinic Acid Combined with Dexamethasone in Management of Newly Diagnosed ITP	Recruiting	Immune thrombocytopenia	Drug: glycyrrhetinicacid|Drug: dexamethasone	Sustained response to ITP treatments|Evaluation of platelet response	Shandong University	Phase 4	30	10 June 2021
NCT00384384	Glycyrrhetinic Acid-Effect on Serum Potassium and Insulin Resistance in Dialysis Patients	Completed	End-stage renal disease	Drug: oral 18B glycyrrhetinic acid versus placebo		University Hospital Inselspital, Berne	Phase 2	24	7 April
NCT00759525	The Role of Mineralocorticoid Receptors in Vascular Function	Completed	Apparent mineralocorticoid excess (AME)	Drug: glycyrrhetic acid|Drug: placebo	Forearm blood flow	Brigham and Women’s Hospital	Phase 2|Phase 3	15	9 September
NCT02939144	An Investigation into the Effect of Liquorice Ingestion on the Salivary Cortisol to Cortisone Molar Ratio	Completed	Apparent mineralocorticoid excess	dietary supplement: liquorice	Salivary cortisol/cortisone ratio induced by licorice (glycyrrhetinic acid and its metabolites) ingestion	The Royal Wolverhampton Hospitals NHS Trust	Not applicable	12	6 March 2017

## 6. Conclusions 

In recent years, there has been considerable growth in the demand for natural source chemicals to acquire health products with better biocompatibility, lower toxicity, therapeutic potential, and lower prices for the general public. This study focused on the pharmacological effects of 18βGA. Despite its reputation as a drug with a broad range of pharmacological effects, 18βGA has yet to be thoroughly studied in terms of pharmacodynamics. Focus should be placed on investigating anticancer activity in preclinical and clinical settings. It has been demonstrated that 18-glycyrrhetinic acid possesses anti-inflammatory characteristics, suggesting that it may be helpful in the treatment of a variety of inflammatory disorders. 18βGA seems to be a potential phytochemical for medication development. Extensive and comprehensive research on 18βGA is needed to speed up the use of 18βGA-based medications in clinical settings. Furthermore, genomic, proteomic, and metabolomic studies should be explored to evaluate bioavailability, half-life, adverse responses, and toxicity characteristics.

## Data Availability

Data sharing is not applicable.
